# Contemporary issues and new challenges in chronic kidney disease amongst people living with HIV

**DOI:** 10.1186/s12981-020-00266-3

**Published:** 2020-03-16

**Authors:** Jack Edward Heron, Corinne Isnard Bagnis, David M. Gracey

**Affiliations:** 1 Department of Renal Medicine, Royal Prince Alfred Hospital, Camperdown, NSW Australia; 2 Nephrology Department, Groupe Hospitalier Pitié-Salpêtrière, 47 Boulevard de l’Hôpital, 75013 Paris, France; 3 Central Clinical School, The University of Sydney, Sydney, NSW Australia

**Keywords:** HIV, Chronic kidney disease, Renal failure, Anti-retroviral therapy, Screening

## Abstract

Chronic kidney disease (CKD) is a comorbidity of major clinical significance amongst people living with HIV (PLWHIV) and is associated with significant morbidity and mortality. The prevalence of CKD is rising, despite the widespread use of antiretroviral therapy (ART) and is increasingly related to prevalent non-infectious comorbidities (NICMs) and antiretroviral toxicity. There are great disparities evident, with the highest prevalence of CKD among PLWHIV seen in the African continent. The aetiology of kidney disease amongst PLWHIV includes HIV-related diseases, such as classic HIV-associated nephropathy or immune complex disease, CKD related to NICMs and CKD from antiretroviral toxicity. CKD, once established, is often relentlessly progressive and can lead to end-stage renal disease (ESRD). Identifying patients with risk factors for CKD, and appropriate screening for the early detection of CKD are vital to improve patient outcomes. Adherence to screening guidelines is variable, and often poor. The progression of CKD may be slowed with certain clinical interventions; however, data derived from studies involving PLWHIV with CKD are sparse and this represent an important area for future research. The control of blood pressure using angiotensin converting enzyme inhibitors and angiotensin receptor blockers, in particular, in the setting of proteinuria, likely slows the progression of CKD among PLWHIV. The cohort of PLWHIV is facing new challenges in regards to polypharmacy, drug–drug interactions and adverse drug reactions. The potential nephrotoxicity of ART is important, particularly as cumulative ART exposure increases as the cohort of PLWHIV ages. The number of PLWHIV with ESRD is increasing. PLWHIV should not be denied access to renal replacement therapy, either dialysis or kidney transplantation, based on their HIV status. Kidney transplantation amongst PLWHIV is successful and associated with an improved prognosis compared to remaining on dialysis. As the cohort of PLWHIV ages, comorbidity increases and CKD becomes more prevalent; models of care need to evolve to meet the new and changing chronic healthcare needs of these patients.

## Introduction

Chronic kidney disease (CKD) is one of the most important non-infectious comorbidities (NICMs) seen in people living with HIV (PLWHIV), both in developed countries and in resource-poor settings [1, 2]. The prevalence of CKD in PLWHIV continues to increase, despite highly effective antiretroviral therapy (ART) [3]. While it has long been recognised that HIV infection is a risk factor for CKD, it is important to note that the pattern of kidney disease affecting PLWHIV has changed [4]. Rather than the previously seen HIV-associated renal conditions, or acute kidney injury (AKI) related to illnesses such as opportunistic infections, CKD now is often related to NICMs, particularly diabetes and hypertension [5]. As well, great disparities are evident, with most HIV infections occurring in minorities and in those in resource poor settings, or of African descent [6]. Long-term exposure to ART in an ageing cohort of PLWHIV contributes to the burden of renal disease [7]. These changes have led to new considerations in PLWHIV, including models of care, access to care in resource-limited settings, polypharmacy and geriatric-specific considerations [8]. With the increasing burden of renal disease seen in this patient group, the increasing numbers of PLWHIV requiring dialysis or kidney transplantation deserve special consideration [9]. This review was undertaken to assess the contemporary issues concerning CKD in PLWHIV and to focus on the challenges arising in the delivery of optimal care.

CKD is an important consideration in PLWHIV, both because of its increasing prevalence, and because of its well-documented adverse effects on patient morbidity and mortality [10]. Once established, CKD usually progresses, and may result in end-stage renal disease (ESRD), where a patient becomes dependent on dialysis or kidney transplantation [11]. The progression of CKD may be slowed with clinical interventions, such as weight loss, blood pressure management and treatment of dyslipidaemia or hyperglycaemia [12]. There are few specific data concerning the benefits of these strategies in PLWHIV, and interventional trials of these approaches are required. It may be that guidelines for CKD in patients with HIV need to be different than those in the general population. Also, CKD is associated with much comorbidity, the most important being cardiovascular disease (CVD), which may impact quality of life and survival [13, 14]. In the general population, strategies for the early detection and prompt management of risk factors associated with CKD, have been shown to be beneficial in improving outcomes and preventing the development of CKD [15]. It is assumed that these same benefits would be seen in PLWHIV [16]. Strategies for the prevention of CKD and for the early detection of CKD amongst PLWHIV are a key concern. Studies have demonstrated that PLWHIV are less likely to receive aspirin for primary cardiovascular disease prevention, and that PLWHIV are less likely to achieve lipid-lowering targets on therapy, when compared to their uninfected counterparts [17]. The relative clinical contributions of the HIV itself, of the prolonged exposure to ART and of clinical risk factors, such as ageing, and NICMs are a difficult balance [18].

## Epidemiology of CKD in PLWHIV

PLWHIV have a higher risk of developing CKD than in the general population. As well, in PLWHIV who are diagnosed with CKD, there is a 2 to 20-fold greater chance of developing ESRD, compared to the general population [19, 20]. This may be explained by the preponderance of risk factors for renal disease in this population; both related to the HIV-itself, as well as adverse effects of ART and comorbid conditions. CKD is also associated with increased hospital admissions, particularly in older patients and those with comorbid CVD [21]. World-wide, access to treatment for ESRD is an increasing concern, with CKD moving from the 27th to the 18th leading global cause of death in the past 20 years. The increase in importance of CKD as a global killer was second only to HIV [18]. Concurrently, there has also been a large increase in NICMs. There is overlap in the comorbidities observed with both CKD and HIV, and globally the increase in conditions, such as diabetes and hypertension, are important considerations in the management of both conditions [22, 23].

CKD is seen in PLWHIV with variable prevalence, depending on geographical location. Different formulas used to estimate renal function and different definitions of CKD between studies may also have a significant effect on estimates [24]. A global prevalence of CKD of 6.4% in PLWHIV using the Modification of Diet in Renal Disease (MDRD) formula, or 4.8% using the Chronic Kidney Disease Epidemiology Collaboration (CKD-EPI) formula, has been reported in a systematic review of studies from 60 countries world-wide; however, the African continent demonstrated the highest prevalence of CKD with 7.9% of PLWHIV affected, according to the MDRD formula [17]. Some studies have reported a much higher prevalence of CKD within Africa, with 44.4% affected by CKD in Cameroon using the CKD-EPI formula [25].

The higher prevalence of CKD in PLWHIV in Africa may be explained by the 18 to 50-fold higher risk in those of African descent world-wide developing HIV-related ESRD compared to Caucasians [26, 27]. The susceptibility to the development of kidney disease is primarily due to genetic factors [28]. Black African PLWHIV in the Western and Southern African continent are likely to carry genetic polymorphisms linked to an aggressive form of HIV-associated nephropathy (HIVAN). More recently, these polymorphisms have also been linked to susceptibility to a wider range of renal conditions, such as hypertension-related renal disease and Focal Segmental Glomerulosclerosis (FSGS) in HIV-negative individuals [28, 29]. The apolipoprotein-1 (APOL-1) gene risk alleles G1 and G2, located on chromosome 22, have been strongly associated with the development of HIVAN; in fact, it is possible that these alleles are pre-requisites for its development [30]. The presence of APOL-1 risk alleles also predicts a more rapid progression of CKD, regardless of the underlying renal histopathology [28]. APOL-1 risk alleles are found in 70–80% of persons of African origin. The distribution of APOL-1 variants is closely associated with the prevalence of Trypanosomal infection; African nations with high frequencies of APOL1 risk alleles also have high rates of Trypanosomal infection, suggesting that these alleles underwent positive selection as a defense against infection [28].

Whilst accounting for much of the observed difference in the prevalence of CKD among PLWHIV, other factors contribute, including environmental factors and access to healthcare [31].

The prevalence of NICMs is significantly associated with the prevalence of CKD; the higher the prevalence of NICMs, the higher the prevalence of CKD [18, 32]. However, the increased susceptibility to CKD is more complex, with a contribution of traditional and HIV-specific risk factors for kidney disease [32]. As shown in Table 1, traditional risk factors for CKD include age, diabetes, hypertension, obesity, a history of AKI and cigarette smoking. Importantly, a large proportion of cases of ESRD in PLWHIV may be prevented with interventions to traditional risk factors [32, 33]. As well, HIV-related risk factors include HIV-viral replication, CD4 count nadir, Hepatitis C (HCV) co-infection and ART [34].

**Table 1:** Traditional and HIV-associated risk factors for kidney disease

Traditional risk factors
Age
Ethnicity
Socioeconomic factors
Diabetes mellitus and obesity
Hypertension
Tobacco misuse
HIV-associated risk factors
History of recurrent acute kidney injury
HIV viral replication
Nadir CD4+ T cell count
APOL 1 gene risk alleles
Anti-retroviral therapy
Illicit drug use
Coinfections, esp. Hepatitis B and C

It can be seen that CKD in PLWHIV represents an immense global problem and has significant implications for healthcare systems [18]. The potential to prevent CKD and ESRF in PLWHIV highlights the importance of screening for, and timely management of, risk factors for CKD, as well as the early detection and management of those with established CKD. Many HIV care providers are not currently designed for such interventions, with the primary focus of many clinics being provision of ART and monitoring of HIV-specific indicators [35].

## Detection of CKD

Clinically, CKD is often asymptomatic and is found on routine screening [36]. Guidelines recommend that PLWHIV be screened regularly for CKD to allow for its early detection and management [34, 37, 38]. PLWHIV who are selected for CKD screening, in addition to a full clinical assessment for risk factors for renal disease and a measurement of their blood pressure, should have a urine test for protein and a blood test for serum creatinine to estimate their renal function [39]. It is recommended that PLWHIV have their GFR estimated at least every 6 months and should have either a urinalysis or a quantitative assessment of their urinary protein excretion at least annually. As well, those whose eGFR has declined by 25% or more, or to a level below 60 mL/min per 1.73 m^2^, or who have protein excretion of over 300 mg/day, should be referred for evaluation by a Nephrologist [37]. PLWHIV who are found to have haematuria with any level of proteinuria, or with an abnormal eGFR, should also be referred for assessment. In PLWHIV receiving TDF, more frequent screening for CKD is also recommended, including monitoring the serum phosphate [47]. Adherence to accepted guidelines for screening for CKD in PLWHIV is variable. Current screening practices fall short of suggested guidelines [40–42].

Clinical risk scores for CKD in PLWHIV have also been developed. These scores may be used to determine an individual’s risk of developing CKD, based on known clinical risk factors for CKD, to grade an individual’s risk of developing CKD. These scores may be useful in clinical decision making, particularly in guiding selection of ART in those at risk [43–45].

The measurement of renal function, or the glomerular filtration rate (GFR), is complex and the most commonly used methods are imperfect [46]. The GFR is usually estimated from the measurement of serum creatinine (eGFR). Creatinine is derived from the metabolism of creatine in the skeletal muscle, and from dietary meat intake. It is unreliable at extremes of body mass index, and may also be influenced by other factors, such as diet and concurrent medications; including some ART [24]. In using the serum creatinine to measure the GFR, an estimation equation is used. Commonly used methods include the Cockcroft-Gault equation, the MDRD formula and the CKD-EPI formula [39]. Because of the effect of skeletal muscle mass on serum creatinine, in elderly individuals with low muscle mass, the renal function may be over-estimated, with important implications for drug dosing [47]. Many different studies have evaluated the performance of these methods to estimate renal function in PLWHIV; it seems likely that the CKD-EPI formula is superior to other equations for estimating the GFR [48]. These methods provide results that are clinically useful in PLWHIV [49].

Other more accurate techniques for the measurement of renal function are available; however, these are more complex, expensive, and rarely used outside the setting of clinical trials. A 24-h urine collection can estimate an individual’s creatinine clearance (CrCl); this technique may be useful when determining appropriate drug doses for renally-excreted medications. Other measurements, such as the iohexol clearance, remain accurate even in PLWHIV who are receiving ART known to affect the renal handing of creatinine. In the research setting several molecules, such as cystatin C, have also been investigated as potential markers of renal function [50, 51]. Cystain C and other specific markers of renal function may be considered for use in situations where the serum creatinine is likely to be unreliable, such as in those individuals with extreme body mass indices, or in those on ART which may affect the excretion of creatinine. Cystain C can be influenced by factors such as inflammation, and the iohexol clearance remains the gold standard for measurement of renal function [52]. In resource limited settings, measurement of renal function may not be performed as part of routine assessments in PLWHIV [53]. Despite this, there is evidence to suggest that ART can be delivered safely without laboratory monitoring in sub-Saharan Africa [54].

Proteinuria may be quantitated with a urinary albumin:creatinine ratio (uACR), or a urinary protein:creatinine ratio. (uPCR) In PLWHIV on TDF a uPCR is preferred, instead of the uACR, which primarily detects urinary albumin, rather than other proteins. The tubular proteinuria seen with TDF toxicity is usually comprised of proteins other than albumin [55]. Both the uACR and uPCR may be used simultaneously to attempt to determine the aetiology of proteinuria in a patient treated with TDF [56]. Other urinary molecules, such as retinol binding protein and β-2 microglobulin, have also been used as potential biomarkers of renal tubular function; mainly in clinical trials [57].

## Causes of renal disease in PLWHIV

As discussed in more detail below; the aetiology of CKD in PLWHIV may be related to the HIV-infection and associated viral replication itself or, more commonly, be due to manifestations of a patient’s NICMs, or from side effects of ART [58]. A classification system of CKD in PLWHIV based on the dominant tissue compartment affected in the kidney may be useful, as shown in Table 2 [59]. To differentiate with certainty between the wide-range of different renal pathologies that are possible in PLWHIV a renal biopsy is required, although, not always clinically available or indicated. The clinical risks of performing a renal biopsy must also be considered [60]. As well, even with a renal biopsy, it can be difficult to attribute the causation of HIV in the pathological changes demonstrated [61].

**Table 2:** Classification of HIV associated renal pathology based on tissue compartment affected

Glomerular compartment
HIV-associated nephropathy
Focal segmental glomerulosclerosis
HIV Immune-complex kidney disease
IgA nephropathy
Membranoproliferative glomerulonephritis^a^
Tubulointerstitial compartment
HIV-associated nephropathy
Acute tubular necrosis
Tubulointerstitial nephritis, often drug-related
Diffuse infiltrative lymphocytosis syndrome (DILS)
Vascular compartment
Atherosclerosis
Thrombotic microangiopathy

^a^Seen with hepatitis C co-infection

### HIV-related kidney disease

#### HIV-associated nephropathy

Classic HIVAN manifests as a collapsing glomerulopathy with hypertrophy and hyperplasia of the overlying glomerular epithelial cells and associated prominent tubulointerstitial changes, including tubular microcysts and damage [62]. Classic HIVAN is strongly associated with the APOL-1 risk allele. Clinically, HIVAN is usually seen as a late complication of chronic HIV-infection, in patients with advanced immunocompromise. Cases of HIVAN have also been described during primary HIV infection with HIV, although, this is less common [63]. HIVAN usually presents with nephrotic-range proteinuria and progresses rapidly to ESRF. The kidneys may appear enlarged and echogenic on renal tract ultrasound [64]. Guidelines suggest the prompt commencement of ART in HIVAN [34, 37]. The choice and dose of ART may be affected by the presence of HIVAN [62]. Corticosteroids have also been used; however, their role is less clear, and their use can be associated with a wide-range of side-effects [65]. Angiotensin converting enzyme inhibitors (ACEi) and angiotensin receptor blockers (ARBs) may also be used.

Non-collapsing FSGS is now more commonly seen than classic HIVAN at renal biopsy [5, 64, 66]. In these patients, the viral load is usually undetectable and they are usually receiving ART. The histological findings may be difficult to distinguish from secondary FSGS. Causality is presumed to be related to HIV when no other secondary cause of FSGS is demonstrated. Effacement of the foot processes may been seen in both the classic collapsing and in the non-collapsing form; although, it is usually less prominent in those with the non-collapsing form [64]. Treatment of non-classic FSGS is usually with ACEi or ARBs; most of these patients are already receiving ART [67].

#### HIV immune complex kidney disease

Numerous forms of immune complex kidney disease may also be seen in PLWHIV. The term HIV immune complex kidney disease (HIVICK) is used to describe these conditions; however, because of the lack of certainty regarding causality of HIV and because of the heterogeneous nature of these conditions, a specific description of the pattern of immune complex deposition in the setting of HIV may be preferred [59, 68]. These conditions include a lupus-like nephritis with negative autoimmune serologies and no other clinical signs of lupus [69]. As well, IgA nephropathy, membranous and membranoproliferative disease have all been described [70, 71]. Membranoproliferative glomerulonephritis may be seen in PLWHIV who are co-infected with hepatitis C virus [72, 73]. The deposition of IgA directed against HIV antigens has been described [70]. Clinically, these conditions have heterogeneous presentations; there may be microhaematuria, a variable degree of proteinuria and impairment of renal function. Immune complex disease benefits from ART and the prognosis appears to be favourable, compared to HIVAN. Consideration of the type and dose of ART used is important [74, 75].

#### Tubulointerstitial conditions

Less commonly, the tubulointerstitium may be involved in an abnormal immune response to HIV. Diffuse infiltrative lymphocytosis syndrome (DILS) is seen rarely in patients who develop a hyperimmune reaction against HIV, and can affect multiple organ systems [76]. DILS is managed by the initiation of ART, although, corticosteroids have also been used [77]. As well, the immune reconstitution inflammatory syndrome may be seen within the kidneys after the initiation of ART. This condition is usually managed by treatment directed to the specific pathogen underlying the response, although, corticosteroids may also be useful [78].

#### Vascular conditions

Progressive vascular disease may be seen in PLWHIV as a direct effect of the HIV on blood vessels within the kidney and is associated with dyslipidaemia and chronic inflammation [79]. This atypical atherosclerosis may be seen as a result of pathological lipid metabolism in the setting of an abnormal immune response [80]. This process is usually seen those with a high burden of risk factors for arteriosclerosis [81]. Abnormal carotid intimal-media thickness has been demonstrated in ART-naïve children without additional risk factors [82]. Management is focused on modifiable risk factors. Type of ART is also an important consideration [14].

Thrombotic microangiopathy is now seen uncommonly; it is as a result of endothelial dysregulation. The condition manifests clinically with thrombocytopaenia, a microangiopathic anaemia and multisystem organ dysfunction, including AKI [83]. It is usually seen in those with high-level viral replication, not on ART. Treatment of this condition may include commencement of ART, plasma exchange and other immunosuppressive agents [84].

### CKD related to viral coinfections

Hepatitis C and B (HCV, HBV) are the two most common coinfections seen in PLWHIV [85]. Both HCV and HBV are associated with CKD, and both may cause infection-associated glomerulonephritis [86]. This can lead to a challenging differential diagnosis of CKD in this population [87]. Worldwide, around 25–30% of PLWHIV are coinfected with HCV and around 5–10% of those with HCV are coinfected with HIV [87]. In CKD with both HCV and HBV coinfection the management is directed at control of viral replication of both conditions. For HCV, direct-acting antivirals (DAA) have revolutionised the treatment of HCV, with selected regimens demonstrating cure in over 95% of HIV/HCV coinfected individuals using short treatment durations of 8–12 weeks [88]. For HCV/HIV coinfection regression of renal lesions has been seen with successful anti-viral management of both conditions [87].

### CKD related to non-infectious co-morbidities

As in the general population, the commonest causes of CKD in PLWHIV are related to NICMs [89]. Hypertension, vascular disease and diabetes are most important causes of CKD in this group [90, 91]. HIV is associated with an increased risk of type 2 diabetes in most series; it is reported to be around four times more prevalent than in the general population [92, 93]. Type 2 diabetes has a greater prevalence and poorer treatment outcomes in PLWHIV compared to the general population [94]. As well, HIV is associated with progressive diabetic nephropathy in animal models [95]. Similarly, hypertension represents a major clinical problem in PLWHIV; in resource-poor settings this hypertension is often recognised and untreated [96]. Secondary FSGS may be related to hypertension; however, there may also be overlap with an HIV-related effect on the glomerulus [96]. Management of these conditions is important to try and slow progression of CKD. Many HIV care services were not designed to address NICMs [97, 98]. The management of comorbidities was identified was a main challenge in meeting the needs of PLWHIV in Australia [99]. This challenge is even greater in resource-limited settings [100].

### Renal effects of ART

Data suggest a role for specific ART medications in contributing to the risk of CKD in PLWHIV [101]. The risk of CKD relates not only to the use of specific ART medications, but also to an individual’s comorbid conditions, and advancing age [102, 103]. CKD and risk factors for the development of CKD effects the choice and dosing of ART [37]. The access to screening tests and the availability of ART may also effect which medications are used [104]. Guidelines recommend the immediate initiation of ART in those diagnosed with HIV. This results in improved life expectancy, with a longer cumulative exposure to ART medications [1]. ART is also important in reducing HIV-related kidney disease [58].

Antiretroviral agents can cause direct toxicity to the kidney, in particular, tubular dysfunction, interstitial nephritis and renal calculi [105]. Recurrent episodes of AKI are a known risk factor for the development of CKD [106]. The management of these conditions is usually supportive; assessment of the type of ART used in these situations are important considerations [107]. The effects of ART on the kidneys are discussed in more detail below.

### Tenofovir disoproxil fumarate

TDF is widely prescribed and is a very effective anti-retroviral agent. TDF is excreted by the kidneys. Dose adjustments are required in those with significant CKD [108]. TDF is associated with proximal renal tubular dysfunction, manifest primarily as non-nephrotic proteinuria and phosphaturia; around 1–2% of all patients on TDF will need to stop treatment because of tubulopathy. In cases of severe tubulopathy there may be severe biochemical abnormalities, such as those seen with the Fanconi syndrome, osteomalacia and acute kidney injury [109]. The Fanconi syndrome is uncommon, and includes renal glycosuria, aminoaciduria, phosphaturia and renal tubular acidosis.

TDF has also been associated with CKD [110].

The precise mechanisms of TDF-associated nephrotoxicity are complex; it is associated with acute tubular necrosis of proximal renal tubular cells and with abnormally enlarged mitochondrial within these cells [111]. In experimental models, tenofovir has been shown to accumulate within tubular cells and induce mitochondrial toxicity [112]. Genetic polymorphisms in proximal renal tubular transporters may account for individual susceptibility to these effects [113]. Risk factors for TDF-nephrotoxicity include increased age, lower baseline renal function, duration of exposure and the concurrent use of ritonavir-boosted protease inhibitors (PIr) [106, 114, 115]. The effect of cumulative exposure to TDF appears important, with each year of TDF use associated with a 14–33% increase in the risk of declining kidney function [115, 116].

The pharmacoenhancer Cobicistat (COBI) has been shown to increase an individual’s TDF exposure, and may increase the risk of TDF-associated nephrotoxicity [113]. TDF levels may also be increased by several other drug–drug interactions, including with non-steroidal anti-inflammatory agents and the antiviral agent ledipasvir (ref) [113].

It is recommended that PLWHIV be evaluated for their risk of CKD before commencing ART and that those on TDF should be screened regularly for the development of proximal renal tubulopathy, which is often asymptomatic [38]. It is recommended that TDF be replaced by a non-tenofovir drug, or by the newer Tenofovir alafenamide (TAF), if there is documented proteinuria, persistent hypophosphatemia, a progressive decline in GFR with no other apparent cause, or osteopenia or osteoporosis in the setting of a high urinary phosphate excretion [37]. It is also recommended that TDF be replaced, or not used, in PLWHIV with significant proteinuria from any cause, a declining GFR, or a high risk of CKD [37]. The effect of switching off TDF on clinical parameters is variable, with some patients exhibiting progressive CKD, despite the cessation of TDF [113].

In addition to its use in ART, TDF combined with emtricitabine (FTC) may also be used for pre-exposure prophylaxis (PrEP); either daily or on-demand [117, 118]. Both dosing strategies are very effective for the prevention of HIV-infection [119]. Toxicity associated with the use of TDF/FTC remains a consideration, although, its safety profile appears much more favourable amongst the HIV-uninfected population [120]. Indeed, in a recent meta-analysis of adverse effects of TDF/FTC for PrEP, there were no significant differences in serious adverse events between the TDF/FTC group and the placebo arm of these studies [121]. Risk factors for TDF toxicity appear similar both in PLWHIV and those on PrEP; recommendations for screening and monitoring are also similar in both groups (ref) [113]. There are; though, few options for PrEP in individuals with significant CKD; the newer pro-drug TAF may be a safe and effective option [107]. Novel agents and alternative delivery methods are currently under development, and may also be useful for PrEP amongst those with significant CKD [122].

### Tenofovir alafenamide (TAF)

TAF is a pro-drug of TDF, which reaches a higher intracellular concentration in peripheral blood mononuclear cells than TDF, despite maintaining a much lower concentration in the plasma. TAF has been demonstrated to have minimal mitochondrial toxicity in vivo. To date, clinical studies of TAF have demonstrated a much more favourable renal and bone side-effect profile [123]. Possible TAF-associated nephrotoxicity has been reported, although, causation was difficult to establish [124]. Fanconi syndrome has also been reported in patients receiving TAF for the treatment of HIV infection, although causation is not definitive [125]. TAF has been used in PLWHIV and in the general population for the treatment of chronic hepatitis B. TAF is recommended as an option for treatment for PLWHIV who develop TDF-related nephrotoxicity, and in those with significant proteinuria or renal impairment at baseline [123]. Switch studies from TDF to TAF suggest a potential reversion of renal toxicity [126]. No dose adjustment of TAF is required, even in PLWHIV with significant renal impairment. TAF is not recommended for use in those with ESRD, although, it has been safely used previously in patients on haemodialysis. Longer-term safety data for this novel agent are emerging. No specific screening tests are recommended in addition to the usual health checks in PLWHIV receiving TAF [123]. Regardless of type of ART used, all PLWHIV should be assessed regularly for their risk of CKD.

### Protease inhibitors

Protease inhibitors are now used less commonly with the advent of newer ART [127]. The protease inhibitors indinavir (IDV), lopinavir (LPV) and atazanavir (ATV) have been associated with nephrolithiasis, which can manifest as crystalluria, haematuria and loin pain; they may be associated with renal calculi and acute kidney injury [128]. These effects may mandate the discontinuation of these agents [37]. In addition to the withdrawal of offending agents, a specialist assessment for a stone-forming tendency should be undertaken; there is a high recurrence rate of renal stone disease in PLWHIV, often thought to be secondary to metabolic complications.

Cohort studies have also found that several PIrs are associated with the risk of CKD [105]. However, the relative contribution of Pis to progressive CKD has been questioned, particularly in those concurrently receiving TDF [129]. In the D:A:D study, including PLWHIV not receiving TDF, the cumulative use of ATV/r and LPV/r was associated with a higher relative risk of CKD, an increase of 22% and 13%, respectively [110]. There is some evidence to suggest that these changes in renal function may largely be determined by the effects of Ritonavir (RTV) on tubular creatinine transport, rather representing a direct nephrotoxic effect of either ATV or LPV [110, 130]. The reduction in eGFR observed with these agents appears reversible upon their cessation, and does not seem to be associated with advanced CKD or ESRD [110]. Data concerning the association of DRV with CKD is less robust [110]. The use of PIrs is also a known risk factor for TDF-nephrotoxicity. PIs have also been associated with a variety of metabolic adverse effects, including weight gain, which may also predispose to the development of CKD [114, 131].

### Cobicistat

COBI is a pharmacoenhancer and is not nephrotoxic; it is a potent inhibitor of cytochrome 3A (CYP3A), and has no inherent anti-HIV effect. It is used to inhibit the metabolism of other ART to allow them to be dosed in single-daily combination preparations [130]. COBI inhibits the multidrug and toxin extruder protein (MATE-1) in the proximal renal tubule, inhibiting the excretion of creatinine by the kidney, without effecting the actual GFR. The increase in the serum creatinine observed is seen early after commencing therapy, and is not progressive; a continued decline in renal function would raise concerns of a different cause of renal dysfunction [132]. The measurement of GFR in patients on COBI is complicated, as are drug–drug interactions [37]. More recently, once-daily combination therapies, such as Bictegravir (BIC), have become available without need for a pharmacological booster agent [133].

### Integrase inhibitors

The integrase strand transfer inhibitors (INSTIs) include raltegravir (RAL), dolutegravir (DTG) elvitegravir (EVG) and BIC. Weight gain has been reported as a side-effect of INSTIs; however, it is not clear if this is a medication-specific, or a class effect [134]. RAL has been reported to reduce renal function; the mechanism underlying this observation is unclear; however, it does not seem to be related to the tubular excretion of creatinine. Small reductions in renal function were been observed in clinical trials of RAL. Additionally, an association with rhabdomyolysis is possible [130]. Very few adverse renal effects have been reported with EVG; it is usually given with a second agent, such as COBI, to boosts its levels and allow once-daily administration; this may affect the serum creatinine. BIC, similarly, has been associated with very few renal side-effects; no boosting agent is required. Whilst not nephrotoxic, DTG has been associated with an increase in the serum creatinine concentration by inhibiting the organic cation transporter (OCT-2) in the proximal renal tubule, without altering the actual GFR [135]. This can affect the estimation of GFR, with an apparent reduction expected. In clinical trials, a modest, non-progressive, increase in the serum creatinine was observed, usually within the first 1–2 weeks after commencing therapy [130]. In the SPRING-2 study after 48 weeks of follow up, the mean CrCl decreased by 16.5 mL/min in the DTG group, compared to 5.4 mL/min in the RAL group [136]. Similar to COBI, a new set-point for renal function is usually apparent soon after commencing therapy; a continuing decline in renal function would raise concerns of a different cause for renal dysfunction [37]. The OCT-2 co-transporter is also responsible for the renal excretion of some medications, including Metformin, and this is a clinical consideration in diabetics commenced on DTG [135]. DTG does not require dose adjustment in CKD [130].

### An approach to the treatment of renal disease

PLWHIV who have CKD and albuminuria are at particular risk of morbidity and mortality [137]. These individuals should be assessed for reversible factors placing them at risk of progression of renal disease; risk factors should be addressed early. Attention to blood pressure control, treatment of hyperglycaemia, hyperlipidaemia and lifestyle factors, such as weight loss and smoking cessation, are important [37, 138]. Pharmacological control of hypertension with an ACE-i or an ARB should be commenced, particularly if there is proteinuria [139]. The renal function and serum potassium should be monitored in people who commence treatment with these agents. A blood pressure target of under 130/80 mmHg is usually appropriate [140]. Other reversible causes for renal impairment should be sought and underlying renal conditions, such as HIVAN, should be excluded. PLWHIV with CKD or ESRD should be commenced on ART, which improves outcomes. Dose adjustment of medications excreted by the kidneys should be undertaken, and the use of nephrotoxic agents should be avoided [37]. The management of PLWHIV with CKD is usually undertaken in consultation with a Nephrologist.

### Polypharmacy

Polypharmacy and the risks of drug–drug interactions are high in PLWHIV, particularly in the older patient group [141]. These concerns may partly explain the lower rate of non-ART medications used for prevention and treatment of CVD in PLWHIV. This is particularly relevant given the ageing of the PLWHIV cohort, and the associated increasing burden of NICMs [142]. Polypharmacy is also associated with adverse drug events, interactions and poor adherence. Adverse drug reactions are much more frequently seen in PLWHIV, compared to the general population [102]. In recent times, there has been a move to simpler two-drug ART regimens in an attempt to simplify ART and improve tolerability [143].

### Dialysis

The number of PLWHIV on dialysis is increasing with the improved survival seen in this group [67]. Access to dialysis should not be impeded because of an individual’s serological status; particularly taking into account the similar life expectancy of a PLWHIV on ART, compared to that of the general population [144]. The one-year survival rate of PLWHIV on dialysis has improved, although, the rate remains inferior to that of the general population. Evidence suggests similar outcomes between haemodialysis and peritoneal dialysis in PLWHIV [145]. Drug dosing and comorbidities are major issues in this group [67].

### Renal transplantation

Improvements in the long-term prognosis of PLWHIV and studies demonstrating good outcomes with kidney transplantation in PLWHIV have prompted many transplant programs to offer kidney transplantation as standard therapy to those PLWHIV with ESRD as an alternative to remaining on dialysis [146]. Studies have demonstrated a better long-term prognosis in selected patients with well-controlled HIV, compared to remaining on the kidney transplant waitlist. The optimal approach for kidney transplantation in PLWHIV has emerged as an important consideration; particularly, immunosuppressant protocols and infection prophylaxis. As well, the potential for drug–drug interactions between immunosuppressants and ART is well recognised; particularly in those receiving CYP3A inhibitors, such as PIr, who receive Tacrolimus-based immunosuppression [147]. Those receiving PIr and Tacrolimus require a dramatic reduction in Tacrolimus doses to avoid supra-therapeutic Tacrolimus levels post-transplant; Tacrolimus is normally metabolised by CYP3A [148]. It is preferable to modify ART to avoid these drug–drug interactions, before kidney transplantation is undertaken [149]. An increase in the risk of acute rejection demonstrated in this group has meant that Tacrolimus-based immunosuppression is used preferentially, with some transplant centres also using additional induction therapy [150]. Despite the increased risk of acute kidney transplant rejection amongst PLWHIV, outcomes are similar to the non-infected population [151]. However, outcomes in individuals co-infected with hepatitis C remain inferior (ref) [149]. Kidney transplantation in PLWHIV has also been performed from both living and deceased HIV-positive donors, with good outcomes [152].

## Conclusions

PLWHIV are at an increased risk of CKD, which is most often related to NICMs, such as diabetes and hypertension. NICMs are becoming increasingly prevalent as the cohort of PLWHIV ages. As a consequence, models of care need to evolve to meet the new and changing healthcare needs of PLWHIV. CKD in PLWHIV is associated with poorer clinical outcomes, including higher morbidity and mortality, and is strongly associated with CVD. Potentially nephrotoxic ART may be avoided if an individual is at high risk of CKD, or if there is established CKD. Regardless of the type of ART used, all PLWHIV are at risk for CKD and should be routinely screened for risk factors and the presence of CKD. Among PLWHIV who are at high risk of CKD, blood pressure control, treatment of hyperglycaemia, hyperlipidaemia and attention to lifestyle factors, such as weight loss and smoking cessation, are important. Control of established hypertension with an ACE-i or an ARB is indicated, particularly if there is proteinuria. These recommendations are based on evidence from studies in the general population, and further research is required to define the optimal strategies for the prevention and management of CKD among PLWHIV. The number of PLWHIV with ESRD is increasing worldwide; this group should not be denied renal replacement therapy, either dialysis or renal transplantation, based on their HIV status.

## Data Availability

Not applicable.

## Abbreviations

ACEi: Angiotensin converting enzyme inhibitor
APOL-1: Apolipoprotein-1
ARB: Angiotensin receptor blocker
ART: Antiretroviral therapy
ATV: Atazanavir
BIC: Bictegravir
CKD: Chronic kidney disease
COBI: Cobicistat
DTG: Dolutegravir
eGFR: Estimated glomerular filtration rate
EVG: Elvitegravir
DILS: Diffuse infiltrative lymphocytosis syndrome
ESRD: End-stage renal disease
FSGS: Focal segmental glomerulosclerosis
HIVAN: HIV-associated nephropathy
HIVICK: HIV immune complex kidney disease
IDV: Indinavir
INSTI: Integrase strand transfer inhibitors
LPV: Lopinavir
NICM: Non-infectious comorbidity
PLWHIV: People living with human immunodeficiency virus
RAL: Raltegravir
TAF: Tenofovir alafenamide
TDF: Tenofovir disoproxil fumarate
uACR: Urine albumin creatinine ratio
uPCR: Urine protein creatinine ratio
